# Advances in Chemokine Signaling Pathways as Therapeutic Targets in Glioblastoma

**DOI:** 10.3390/cancers13122983

**Published:** 2021-06-15

**Authors:** Ruth M. Urbantat, Peter Vajkoczy, Susan Brandenburg

**Affiliations:** 1 Department of Experimental Neurosurgery, Charité—Universitätsmedizin Berlin, Corporate Member of Freie Universität Berlin and Humboldt-Universität zu Berlin, 10117 Berlin, Germany; peter.vajkoczy@charite.de (P.V.); susan.brandenburg@charite.de (S.B.); 2 Department of Neurosurgery, Charité—Universitätsmedizin Berlin, Corporate Member of Freie Universität Berlin and Humboldt-Universität zu Berlin, 10117 Berlin, Germany

**Keywords:** GBM, chemokine receptors, targeted therapy, antiangiogenic therapy, immunotherapy

## Abstract

**Simple Summary:**

Chemokine signaling is crucial for tumorigenesis, proliferation, angiogenesis, tumor progression and metastasis in glioblastoma. Furthermore, chemokines have an impact on the overall survival of GBM patients that, with a median survival of 15 months, still lack effective treatment. Several chemokine signaling axes, for instance CXCR2/CXCL2/IL-8, CXCR4/CXCL12 and CCR2/CCL2, were investigated in detail and appear to be promising therapeutic targets. The aim was to review novel approaches that target chemokines or chemokine receptors in glioblastoma. Here, preclinical and clinical studies were used to clarify their significance for targeted treatments alone and in combination with other therapeutic applications.

**Abstract:**

With a median patient survival of 15 months, glioblastoma (GBM) is still one of the deadliest malign tumors. Despite immense efforts, therapeutic regimens fail to prolong GBM patient overall survival due to various resistance mechanisms. Chemokine signaling as part of the tumor microenvironment plays a key role in gliomagenesis, proliferation, neovascularization, metastasis and tumor progression. In this review, we aimed to investigate novel therapeutic approaches targeting various chemokine axes, including CXCR2/CXCL2/IL-8, CXCR3/CXCL4/CXCL9/CXCL10, CXCR4/CXCR7/CXCL12, CXCR6/CXCL16, CCR2/CCL2, CCR5/CCL5 and CX3CR1/CX3CL1 in preclinical and clinical studies of GBM. We reviewed targeted therapies as single therapies, in combination with the standard of care, with antiangiogenic treatment as well as immunotherapy. We found that there are many antagonist-, antibody-, cell- and vaccine-based therapeutic approaches in preclinical and clinical studies. Furthermore, targeted therapies exerted their highest efficacy in combination with other established therapeutic applications. The novel chemokine-targeting therapies have mainly been examined in preclinical models. However, clinical applications are auspicious. Thus, it is crucial to broadly investigate the recently developed preclinical approaches. Promising preclinical applications should then be investigated in clinical studies to create new therapeutic regimens and to overcome therapy resistance to GBM treatment.

## 1. Introduction

Glioblastoma (GBM) is the most common malignant brain tumor in adults [1,2] and belongs to the aggressive high-grade gliomas (grade IV). The incidence of GBM ranges from 3.20 to 4.64 per 100,000 people [1,3]. Since 2016, a new WHO classification has been applied [4]. There, GBMs were divided into three groups depending on the status of the isocitrate dehydrogenase (IDH): *IDH*wt GBM, mutated *IDH* GBM and not otherwise specified GBM (NOS, unevaluated status). *IDH*wt GBMs represent 90% of glioblastomas and are mainly primary GBMs that develop de novo in elderly patients. Only 10% of GBM patients exhibit an *IDH* mutation and correspond to secondary GBMs which originate from astrocytic tumors or oligodendrogliomas that occur in younger patients and have a better prognosis. Furthermore, GBMs were described by various molecular biomarkers besides *IDH*, including *MGMT* promoter methylation (O^6^-methylguanine DNA methyltransferase), chromosome 1p/19q deletion, *TERT* (telomerase reverse transcriptase) promoter mutation, *TP53* (tumor protein P53) mutation, *PTEN* (phosphatase and tensin homolog) mutation, *EGFR* (epidermal growth factor receptor) and *PDGFRA* (platelet-derived growth factor receptor A) amplification. Especially, the *MGMT* promoter methylation is often used as a prognostic marker in GBM. A higher *MGMT* promoter methylation leads to a lower *MGMT* expression, supporting a better prognosis of the respective GBM patients [5]. The *MGMT* enzyme repairs the DNA damage caused during temozolomide (TMZ) therapy and therefore is responsible for drug resistance of glioblastoma cells to anticancer treatments [6]. Despite tremendous efforts in the past decades to improve treatment strategies and to overcome the development of resistance, overall GBM patient survival (OS) does not exceed 15 months [7]. The difficulties of treating glioblastoma are based on its biology, exhibiting a high level of vascularization, invasiveness and complex cell composition.

This highly vascularized tumor shows tremendous growth and depends on the formation of new blood vessels [8,9,10]. Activation of numerous angiogenic receptors and upregulation of their respective ligands promote angiogenesis in GBM and thus sustain tumor progression [8,9]. Here, especially the VEGFR/VEGF pathway was extensively studied, leading to the development of several anti-VEGFR/VEGF drugs for GBM treatment, although without significant improvement of survival [8,11,12].

A special feature of GBM is the high infiltration of myeloid cells consisting of resident microglia and peripheral macrophages [13] which make up to 30–50% of the total glioma mass [14]. The number of these tumor-associated microglia/macrophages (TAMs) in glioma was correlated with tumor malignancy [13]. Interestingly, their functions were controversially discussed. Tumor-supportive, immunosuppressive properties (M2 status) of TAMs were frequently determined [15,16], but antitumoral, proinflammatory effects (M1 status) were also described [17]. However, recent studies suggest that proinflammatory as well as immunosuppressive molecules are expressed by TAMs in human and rodent glioblastomas [18,19,20]. Besides TAMs, additionally, CD8^+^ cytotoxic T lymphocytes (CTLs), CD4^+^ T helper cells (Th1), regulatory T cells (Treg) and natural killer (NK) cells infiltrate glioma tissues [21]. Thus, immunotherapies for glioblastomas were established [22]. Nevertheless, the development of such immunotherapies is challenging in GBM, due to the lack of lymphatic involvement, the need to overcome the blood–brain barrier [23] and the immunosuppressive tumor microenvironment [22,24].

Another cell population that occurs in glioblastoma tissues are glioma stem cells (GSCs). GSCs have the capability for self-renewal and differentiation to form a tumor [25]. These GSCs develop by differentiation of tumor cells following radio- or chemotherapy [26] and by malignant transformation of neural stem cells [27]. Importantly, GSCs are more resistant to drug administration than other tumor cells elucidating their relevance for development of resistance and GBM recurrence [28].

Consequently, despite new therapeutic approaches, including antiangiogenic treatment, tumor treating fields (TTF) and immunotherapies, OS has only marginally improved for GBM patients in recent years [11,12,29,30,31,32,33]. Therefore, further efforts were made to develop novel strategies to fight glioblastoma, including targeting chemokines and chemokine receptors based on their impact on GBM biology and progression.

Chemokines are small proteins known as the largest subfamily of cytokines [34]. The name “cytokine” derives from the Greek “kinos” which translates to “movement”. Their traditional role is to mediate the migration of many distinct cell types throughout the body [35,36]. Depending on the position of the two cysteines (C) near the N-terminus, chemokines can be divided into different subfamilies: CC, CXC, CX3C and XC [37,38,39]. Chemokine signaling is mediated through their cognate receptors, which are typically G protein-coupled [37,39]. So far, more than 50 chemokines have been identified in humans [39,40]. A variety of cells including monocytes, TAMs [41,42], T lymphocytes, neutrophils, fibroblasts, neural, endothelial [43,44,45], epithelial and tumor cells secrete these molecules [46,47]. Chemokine receptors are preferentially expressed by immune cells including TAMs, and also by brain endothelial cells and tumor cells [37,41,43,44,47,48,49]. It was demonstrated that various chemokines and their receptors are expressed by cancer entities including breast cancer, ovarian cancer, cervical cancer, prostate cancer and glioma [34]. Recent studies have shown that chemokine receptors CXCR2, CXCR4, CXCR6, CXCR7, CCR5, CX3CR1 as well as the chemokines CXCL2, IL-8, CCL2 and CX3CL1 are highly expressed in GBM [49,50,51,52,53,54,55,56,57,58,59,60]. Therefore, over the past decade, many of these signaling pathways have been investigated as potential targets in GBM treatment.

**Figure 1:**
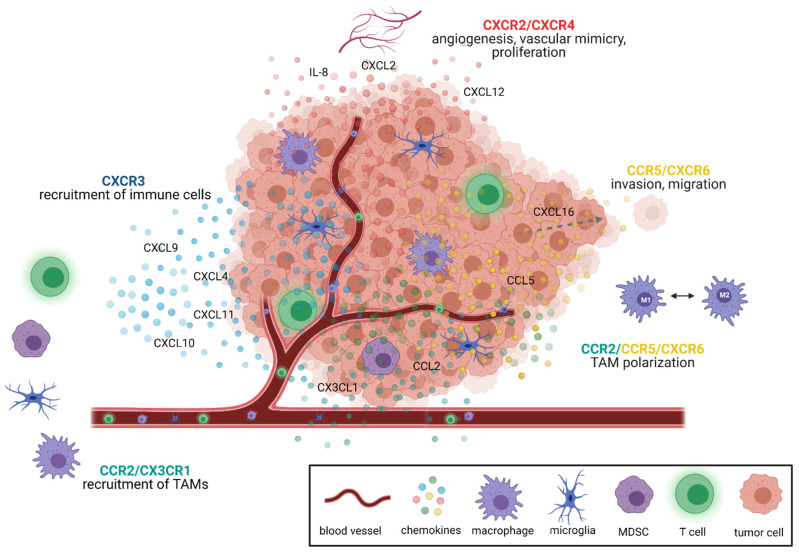
The most important functions of various chemokine signaling pathways in GBM. Chemokine signaling axes shape the tumor microenvironment by mediating many different processes. For instance, CXCR2 and CXCR4 signaling is especially important for tumor angiogenesis, proliferation and the formation of vascular mimicry. CCR5 and CXCR6 mediate invasion and migration in GBM. In addition, CCR5 and CXCR6 alongside CCR2 signaling influence TAM polarization. Apart from CCR2 signaling, CX3CR1 is involved in the recruitment of TAMs. Other immune cells are mainly recruited to the tumor by CXCR3 signaling. Chemokines are depicted as dots and color-coded matching the color of their corresponding receptor. Created with BioRender.com.

Within the tumor, chemokines support many tumor-sustaining processes such as cell proliferation, tumor growth, angiogenesis and metastasis, and thus modify the tumor microenvironment [34,39,40,49,55,61]. The different chemokine signaling pathways relevant for glioblastoma were recently reviewed in detail by Groblewska et al. [39]. In brief, especially CXCR2 and CXCR4 mediate tumor angiogenesis [39,62,63]. CXCR3 and CCR2 signaling leads to the recruitment of tumor-promoting immune cells, e.g., of TAMs, T cells and myeloid-derived suppressor cells (MDSC) [34,37,39,64]. Furthermore, CCR2, CCR5 and CXCR6 signaling modulates TAM polarization [39,65,66]. The other main functions of CCR5 and CXCR6 signaling include proliferation and invasion of glioma cells [39,65,66] (Figure 1). Thus, these chemokine/chemokine receptors served as the basis for the development of novel antiangiogenic and immunotherapeutic strategies in GBM treatment.

In this study, we aimed to review preclinical and clinical studies regarding the CXCR2/CXCL2/IL-8, CXCR4/CXCL12/CXCR7, CCR2/CCL2, as well as the less investigated CXCR3/CXCL4/CXCL9/CXCL10/CXCL11, CXCR6/CXCL16, CCR5/CCL5 and CX3CR1/CX3CL1 signaling pathways. We demonstrated different therapeutic approaches as targeted therapies, in combination with temozolomide (TMZ, the standard-of-care chemotherapeutic agent) and radiotherapy, antiangiogenic agents as well as immunotherapies.

## 2. Chemokine Signaling Pathways as Therapeutic Targets in GBM

### 2.1. Targeted Therapies in Chemokine Signaling

Targeted therapies are an important step towards precision medicine and overcoming therapy resistance. While one therapy might help one patient, it might not help another one. The standard-of-care therapeutic regimens for GBM, established more than a decade ago, evidently fail to prolong patient OS for more than a few months [2,4]. Therefore, targeting chemokine signaling, a highly abundant network within the tumor microenvironment, is a promising approach.

Enhanced expression of CXCR2 and its ligands is associated with malignancy and recurrence in glioma [46,67]. *IL8, CXCL2, CXCR1* and *CXCR2* are expressed by various GBM tumor cell lines as well as by tumor cells and endothelial cells in GBM patients [46,49,60,61,68,69]. Raychaudhuri and Vogelbaum showed that the signaling pathway is involved in the aberrant activation of NF-κB [61]. Blocking IL-8 by a neutralizing antibody as well as treatment with a small interfering RNA targeting *IL-8* reduced GBM cell invasiveness in vitro [61]. This was later confirmed in a 3D spheroid-based coculture model of patient-derived GBM cells with brain endothelial cells [70]. There, the authors also found that tumor cell invasion was additionally enhanced by endothelial cells which stimulated cancer stem cells through IL-8 signaling. Blocking IL-8 resulted in reduced spheroid formation in vitro and tumor formation in vivo in an orthotopic mouse model using human GBM cells [70].

Mesenchymal stem cells (MSC) showed multipotent differentiation characteristics in vitro and can be isolated from the bone marrow, adipose tissue and the umbilical cord as described before [71]. Due to their unique capacity of self-renewal and multipotency, they have been widely investigated in cell-based therapeutic approaches [72]. However, there are conflicting data on the effect of MSCs in tumor treatment. While some studies report protumoral effects [73,74,75], others demonstrate antitumoral efficacy of MSCs which have been described in detail in multiple reviews [75,76,77]. Controversial findings regarding MSCs and CXCR2 signaling were demonstrated by Bajetto et al. Interestingly, their study found that cocultivation of glioma stem cells (GSCs) with MSCs resulted in reduced proliferation. However, when treated with an MSC-conditioned medium, GSCs exhibited increased proliferation due to several chemokines within the MSC-conditioned medium, including IL-8, CXCL1 and CXCL5. These chemokines mediate their functions through CXCR2. Proliferation and migration of GSCs in the cultured MSC-conditioned medium was significantly reduced by blocking CXCR2 with SB225002, a highly specific CXCR2 antagonist [75]. Furthermore, Hasan et al. demonstrated the importance of CXCR2/IL-8 signaling in tumor growth and therapy resistance to temozolomide in a patient-derived xenograft (PDX) GBM model [46]. IL-8 expression in GBM patients correlated with reduced progression-free survival (PFS) and was elevated in recurrent tumors. The authors found that IL-8/CXCR2 signaling supports the self-renewing capacity of GBM cells and increased the expression of glioma-initiating cell markers [46]. Moreover, IL-8 knockdown significantly enhanced OS in tumor-bearing mice and augmented the therapeutic efficacy of TMZ [46].

Apart from blocking antibodies, antagonists and gene knockdown, Hübner et al. targeted a network of proinflammatory genes. In their innovative approach, using microRNA-93 (miR-93) which directly regulates glioma cell gene expression of HIF-1α and MAP3K2, cytokines and chemokines like IL-8 and also indirectly targets CXCL5 and COX-2, they showed various functions of this specific miRNA [78]. Interestingly, miR-93 is downregulated in GBM tissue and GBM cells, compared to normal brain tissue. Furthermore, re-expression of miR-93 decreased proliferation, migration and angiogenesis in vitro [78].

CXCR3 signaling mediated by CXCL9, CXCL10, CXCL11 and CXCL4 plays a major role in inflammation and immunopathology as well as in prohibiting angiogenesis in gliomas [37,39,79]. CXCR3 is widely expressed by Th1 cells, CD8^+^ T cells and NK cells [34,37], but also by tumor cells [80,81,82,83,84]. While little is known about CXCL4 expression in GBM, CXCL9, CXCL10 and CXCL11 are expressed by tumor cells [83,84,85]. Additionally, CXCL9 and CXCL10 are also expressed by M1-polarized TAMs [86] and CXCL11 can be found on tumor vasculature [84]. Targeting this pathway as an immunotherapeutic approach is discussed further on in this review. However, downstream signaling via CXCR3 can also promote cell proliferation, survival and migration or inhibit these, depending on the receptor isoforms [80,81,82,87]. CXCR3-A executes the previously described functions while CXCR3-B executes the latter [80,81,82,87]. Both variants are expressed at low mRNA levels in glioma cells [82]. Wang et al. showed that genetically engineered upregulation of long non-coding RNA 135528 (Lnc135528) promotes tumor regression by upregulating CXCL10 and the JAK/STAT signaling pathway in vivo [83]. However, a different study has shown upregulation of CXCR3 and CXCL10 in GBM tumors on protein expression levels, compared to diffuse astrocytoma tumor samples [85]. Nevertheless, it is possible that gene and protein expression levels are differentially regulated.

The CXCR4/CXCR7/CXCL12 signaling pathway has been extensively studied in recent years and is one of the best-evaluated chemokine signaling pathways in the glioblastoma microenvironment. Among its various functions, CXCR4 signaling is linked to glioma cell invasion, proliferation, tumor progression and angiogenesis [88,89,90,91,92]. As many other chemokine receptors, CXCR4 is overexpressed in glioma-initiating cells/GBM tissue and associated with a reduced PFS and OS [53,93]. Furthermore, irradiation induces the local expression of CXCL12 which promotes tumor recurrence [94,95]. Another mechanism of tumor recurrence is the resistance to TMZ treatment which could be promoted by the upregulation of FOXM1 mediated through CXCR4/CXCL12 [96]. Apart from tumor and endothelial cells, CD4^+^ T lymphocytes express CXCR4 [39,97,98].

AMD3100, also known as plerixafor, is an FDA-licensed CXCR4 antagonist which inhibited migration of glioma cells in vitro under normoxic and hypoxic conditions [99,100]. Furthermore, it reduced chemotaxis, survival and proliferation of glioma cell lines and neurospheres [101,102]. In a 3D vascular environment model as well as in transwell assays, AMD3100 additionally inhibited invasion [91,103]. These positive effects of AMD3100 were reproduced in several mouse models [99,101,104,105]. AMD3100 led to inhibition of tumor growth [99,101], possibly by increasing apoptosis in orthotopic PDX mouse models [101]. Another study found that when treating a variety of GBM cells in vitro, the effective dose of combined AMD3100 with 1,3-bis(2-chloroethyl)-1-nitrosourea (BCNU, a chemotherapeutic agent) was dependent on CXCR4 protein expression [104]. Therefore, higher protein levels also required higher treatment doses to achieve the same response [104]. In their study, Redjal et al. then confirmed the in vitro findings in a PDX GBM mouse model. There, combining AMD3100 with BCNU led to tumor regression by increasing apoptosis and decreasing proliferation [104]. Cornelison et al. recently demonstrated that convective flow forces within the tumor tissue may increase GBM invasion dependent on CXCR4 [105]. Hence, application of AMD3100 effectively inhibited convection-induced invasion [105]. In summary, these studies show that AMD3100 is able to successfully inhibit crucial tumor-driving effector mechanisms of CXCR4/CXCL12 signaling in vitro and in vivo.

There is a vast number of additional CXCR4 inhibitors including CPZ1344, AMD3465 and Peptide R that reduced tumor growth [106,107,108] and inhibited migration and angiogenesis of glioma cells [106]. CXCR7, an alternative receptor to CXCL12, is also highly expressed in glioma cells, endothelial and microglial cells and mediates antiapoptotic effects [56]. Additionally, high expression of CXCR7 is independently correlated with poor OS in GBM patients [109]. Among the CXCR7 antagonists are CCX771 and CCX733, of which CCX771 inhibited glioma cell proliferation and invasion [110] and CCX733 reduced proliferation and antiapoptotic effects in vitro [56,111]. These results were underlined by suppression of CXCR7 through small interfering RNA (siRNA) which led to decreased glioma cell proliferation, migration, and invasion as well [110]. Utilizing CXCR4 and CXCL12 antibodies markedly inhibited glioblastoma cell proliferation and thus tumor growth [112,113]. Overall, therapeutic approaches blocking the CXCR4/CXCR7/CXCL12 signaling pathway are highly effective independent of whether the ligand or the receptors are being blocked.

Apart from pharmacological approaches, some researchers focused on different microRNAs and how to best exploit their antitumoral functions in GBM. The expression of miR-9 directly targeted CXCR4 signaling in GBM; it functioned as a tumor suppressor by inhibiting the migratory capacity of GBM cells in vitro [53]. Another miRNA, miR-663, negatively regulated CXCR4 expression, thus compromising the proliferative and invasive capacities of GBM cells in vitro [114]. Overexpression of miR-663 in combination with AMD3100 reduced GBM growth in an orthotopic mouse model while simultaneously prolonging the survival of tumor-bearing mice [114]. CXCR4 signaling and its effect on tumor cell stemness, self-renewal and infiltration can also be inhibited by the miR-302–367 cluster which consists of five different microRNAs (miR-302a, miR-302b, miR-302c, miR-302d, miR-367) [115]. In a later study, Fareh et al. found that this tumor suppressive cluster miR-302-367 is transferred between cells via exosomes [116]. Furthermore, the expression of the miR-302-367 cluster in glioma stem cells acted as a tumor suppressor in the paracrine manner in an orthotopic PDX mouse model [116].

A different approach that utilizes CXCR4 overexpression in glioma cells without directly interfering in the pathway was recently proposed by Gascon et al. [117]. The use of nanoparticles as a delivery system for CXCL12 to GBM cells was established in vitro [117]. There, authors enclosed CXCL12 in nanoparticles which, upon delivery, released CXCL12 to GBM cells, without promoting proliferation but keeping its chemotactic capabilities [117]. The aim for the future is, to inject these CXCL12-carrying nanoparticles into a hydrogel which could be used to fill up the cavity after tumor resection, thus attracting disseminated tumor cells. These tumor cells would then be targeted by standard therapeutic approaches while having the benefit of not affecting the surrounding healthy brain tissue [117].

Another pillar of novel therapeutic approaches is cell-based, specifically, stem cell-based therapy. There are conflicting data on the advantages and disadvantages of this specific therapeutic approach. Whereas some studies showed that migration of CXCR4-overexpressing human adipose-derived stem cells and human mesenchymal stem cells to the tumor resulted in reduced tumor volume and prolonged survival of tumor-bearing mice [118,119], Pavon et al. showed that human umbilical cord blood MSC also exhibit protumoral functions [120]. In their rodent model, MSCs promoted tumor growth after infiltrating the tumor [120]. In summary, there is a variety of therapeutic approaches targeting the CXCR4/CXCR7/CXCL12 axes including antagonists, blocking antibodies, nanoparticles, stem cell-based therapies, which all showed remarkable effects in vitro and in preclinical in vivo models.

Another important signaling pathway in GBM is mediated by CCR5 and CCL5. Key functions of both the receptor and the ligand have been demonstrated. For instance, high expression of CCR5 and CCL5 correlates with a survival disadvantage of GBM patients [58]. Furthermore, CCR5/CCL5 signaling is known to drive tumor heterogeneity, the formation of cancer stem cells and therefore promotes cancer invasion and metastasis in vitro [58,121]. *CCR5* downregulation significantly reduced tumor growth in a glioma xenograft mouse model [58]. However, in another study, *CCR5* deletion had no effect on the survival of tumor-bearing mice [122]. Blocking CCR5 on microglia in vitro with maraviroc, an FDA-approved drug for HIV treatment, suppressed microglial migration and reduced M2 phenotype-specific markers arginase 1 and interleukin 10 while inducing the expression of M1 phenotype markers like nitric oxide and interleukin 1β [65]. In patient-derived primary glioblastoma cells as well as in glioma stem cells, maraviroc strongly inhibited CCR5-mediated invasion in vitro [57]. CCR5 is also constitutively expressed by T lymphocytes [97]. The immunosuppressive microenvironment in GBM will also be discussed in this review. Nevertheless, GBM is only infiltrated by a low number of CD8^+^ T cells, however, a subset of activated CD8^+^ T cells has been shown to be enriched with CCR5 and is associated with disease progression [123].

Glioma cells express both CX3CR1 and CX3CL1 at mRNA and protein levels [59,124]. Furthermore, *CX3CL1* expression correlates with glioma grade and serves as a prognostic marker for glioma patient OS [59]. Analysis based on The Cancer Genome Atlas (TCGA) and the Chinese Glioma Genome Atlas (CGGA) databases showed that high levels of *CX3CL1* positively correlate with patient OS in glioma [125]. Sciume et al. demonstrated that endogenous CX3CL1 negatively regulated the invasiveness of three individual glioma cell lines in vitro [124]. In addition to that, blocking CX3CL1 with a monoclonal antibody increased glioma cell invasion and reduced tumor cell aggregation [124]. Apart from tumor cells, TAMs [126] and NK cells [125], which account for up to 50% of the cellular components of GBM [14,127], express CX3CR1. They mediate important processes including migration, accumulation of TAMs, adhesion and angiogenesis during malignant transformation [125,126,128].

A variety of these promising therapeutic approaches has been evaluated in detail in preclinical in vivo models and clinical phase I and II studies which will be discussed in the respective chapters. Firstly, we reviewed the combination with the standard-of-care therapy; secondly, the combination with antiangiogenic agents; and lastly, immunotherapeutic approaches.

### 2.2. Combination of Targeting Chemokine Receptors/Chemokines with the Standard-of-Care Therapy

The current standard-of-care for GBM was established more than 15 years ago and is known as the Stupp protocol, which includes maximal resection and radio-chemotherapy followed by six cycles of temozolomide [1,2]. Grand total resection (>98%) has a significant impact on patient OS [129,130,131]. As GBM is a highly infiltrative tumor, it is hardly possible to reach 100% resection; thus, radiochemotherapy is crucial to target the remaining tumor cells. However, GBM prognosis, with the median OS of 15–18 months, remains dismal [130,131].

The CXCR4/CXCR7/CXCL12 signaling pathway plays an important role in proliferation, differentiation, cell survival and chemotaxis in glioblastoma [40,89]. NOX-A12, also known as olaptesed pegol, a CXCL12 inhibitor, has been evaluated in a GBM rat model after irradiation [132]. There, Liu et al. demonstrated dose- and time-of-treatment-dependent prolonged survival in rats treated with NOX-A12 [132]. Interestingly, targeting CXCL12 inhibited both signaling pathways through CXCR4 and CXCR7 [132]. NOX-A12 alone after irradiation exceeded the effect of NOX-A12 in combination with TMZ or TMZ alone after whole-brain irradiation (WBIR) on rat OS [132], once more supporting the outstanding effect of blocking CXCR4/CXCR7/CXCL12 signal transduction. Therefore, NOX-A12 is currently being evaluated in a clinical phase I/II study in combination with irradiation in patients with newly diagnosed GBM with either unmethylated MGMT promotor status or patients unsuitable for surgery.

These findings are emphasized by the effect of three alternative CXCR7 inhibitors CCX771, CCX662 and X7Ab which led to tumor regression [55,84], blocked tumor recurrence [84] and increased survival [55] in several mouse models. Furthermore, CXCR7 plays an important role in cancer stem cell activity in vitro [84]. The antitumoral functions are likely explained by enhanced NK cell efficacy as well as increased phagocytic activity of TAMs [55]. Moreover, combining X7Ab with temozolomide in two orthotopic GBM mouse models had a significant effect on mouse survival compared to X7ab or TMZ alone and the control group [55]. Adding to that, the combination therapy allowed for the TMZ dose to be reduced while still showing significant tumor reduction [55]. If this could be reproduced in clinical studies, it would ultimately lower adverse effects for GBM patients.

Due to the groundbreaking achievements in preclinical studies with AMD3100 blocking CXCR4/CXCL12 signaling, its efficacy in the first clinical studies is being investigated. In 2013, a then 66-year-old male patient received AMD3100 as part of his first-line therapy [133]. The patient underwent gross total resection and radiochemotherapy, followed by adjuvant temozolomide in combination with AMD3100 [133]. AMD3100 was administered subcutaneously at 0.24 mg/kg of body weight once a week [133]. Additionally, lapatinib, a multikinase inhibitor, niacinamide and metformin were administered [133]. After 12 months of this combination therapy, temozolomide and lapatinib were discontinued [133]. In 2016, 30 months after his initial diagnosis, the patient still showed no clinical or radiological signs of recurrence while still on AMD3100, niacinamide and metformin [133]. The authors explained this ongoing remission by inhibition of the CXCR4-dependent neovascularization after irradiation in GBM. Bone marrow-derived cells (BMDC), specifically, monocytes and endothelial cell progenitors, account for this neovascularization, and their migration is mediated through CXCR4 signaling which will be elaborated on in the following chapter [84,94,132,134]. A recent phase I/II clinical study (NCT01977677) with a total of 29 patients confirmed this hypothesis [135]. There, nine patients with newly diagnosed GBM were included in their phase I study with the aim to evaluate the toxicity profile of AMD3100, while the aim of the phase II study including 20 patients was to assess the efficacy of AMD3100 on PFS and OS [135]. There, 200–400 µg/kg/day AMD3100 were administered intravenously after chemoirradiation for four weeks, followed by adjuvant temozolomide therapy. AMD3100 exhibited no severe side effects [135]. The PFS of 14.5 months and OS of 21.3 months were significantly prolonged compared to the average PFS and OS in GBM patients receiving the standard Stupp protocol [2,4,135]. Interestingly, vasculogenic parameters like the cerebral blood flow volume within the irradiation field were significantly decreased after one (−18%) and six months (−31%) in the patients receiving AMD3100 compared to the patients receiving the standard-of-care regimen [135]. In the patients who developed recurrence, a distinct pattern was observed. Interestingly, recurrence occurred outside of the irradiation field in almost half the cases which is recognizable as usually fewer than 20% of patients treated with radiochemotherapy develop recurrence outside of the irradiation field [135,136]. This underlines the efficacy of the combined approach within the irradiation field in this study, and poses the question whether WBIR would have a different effect in combination with AMD3100 and temozolomide. A clinical phase II study (NCT03746080) currently investigates the efficacy of WBIR with standard temozolomide and AMD3100 in patients with newly diagnosed glioblastoma. Apart from PFS, OS and toxicity, one of the outcome measures is also to assess the out-of-field occurrence of recurrence. However, to finally assess treatment efficacy of combined treatment with AMD3100, further studies with larger patient cohorts are warranted.

Other therapeutic approaches include unconventional therapies like omeprazole, a proton-pump inhibitor. Omeprazole inhibits GBM cell invasion and migration through acceleration of tumor-suppressive properties of the aryl hydrocarbon receptor (AhR) which is expressed in GBM in vitro and in a subcutaneous PDX glioma mouse model [137]. AhR suppresses gene expression of *CXCL12* and *CXCR4* which was enhanced by treatment with omeprazole [137]. The effect of combining omeprazole with TMZ in vitro compared to either treatment alone significantly suppressed migration and proliferation. However, the combination therapy had no additional effect on glioma invasion [137].

**Figure 2:**
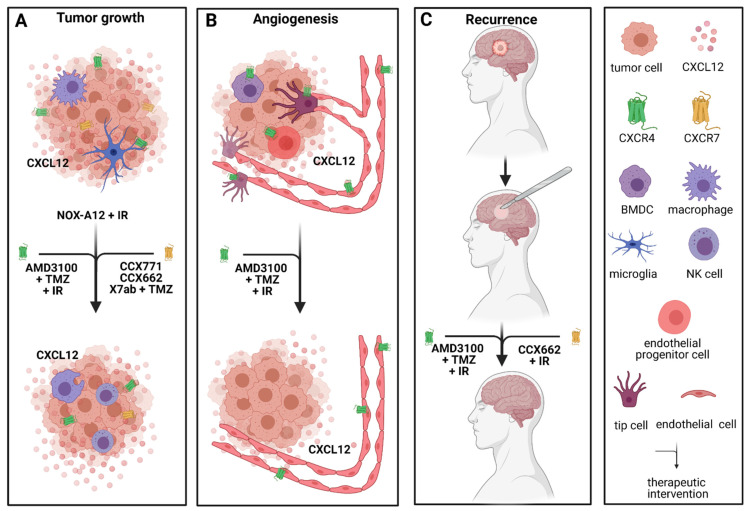
Effects of targeting CXCR4 and CXCR7 signaling in combination with the standard-of-care treatment in preclinical in vivo models and in phase I and phase II studies. (**A**) Tumor growth is reduced by multiple drugs targeting CXCR4 (depicted in green), CXCR7 (depicted in yellow) and CXCL12 (NOX-A12 + IR). The phagocytic activity of TAMs and efficacy of NK cells is increased. (**B**) Angiogenesis is reduced by anti-CXCR4 treatment. (**C**) Combining the standard-of-care therapy consisting of surgical removal of the tumor, radio- and chemotherapy with anti-CXCR4 or anti-CXCR7 treatment hinders the development of recurrence. TMZ = temozolomide; IR = irradiation. Created with BioRender.com.

Another very interesting approach of combining radiotherapy with CXCR4-targeted therapy was recently introduced by Séhédic et al. Their novel model of a nanocarrier-loaded combined lipophilic thiobenzoate complex of rhenium-188 in the core of a nanocapsule (LNC^188^Re) could potentially reach disseminated tumor cells which often escape therapeutic approaches [138]. In their study, they encapsulated Rh-188 and a CXCR4 blocking antibody (12G5) in a lipophilic nanoparticle which, upon delivery to tumor cells, targets CXCR4 and exposes it to radiotherapy [138] (Figure 2).

### 2.3. Antiangiogenic Approaches Targeting Chemokine Receptors/Chemokines

There are many aspects to antiangiogenic treatment (AAT) in GBM. VEGF and its receptors are abundantly expressed in GBM [139]. Angiogenesis is a complex process. In brief, the formation of new blood vessels is guided by tip cells which form upon activation of VEGFR2 [140]. They are characterized by their position, filopodia and ability to guide the following stalk cells into the new environment [140]. Stalk cells are highly proliferative and help form a new sprout [140]. AAT targeting the VEGF/VEGFR pathway has been evaluated in both preclinical and clinical studies [8,11,12]. However, the promising preclinical effects were only partially reproduced in clinical trials, and patient OS was not improved by targeting the VEGF/VEGFR pathway [11,12,29,30]. Phase III clinical studies combining anti-VEGF treatment, known as bevacizumab, with radiochemotherapy or chemotherapy in newly diagnosed GBM failed to improve patient OS [29,30]. Current clinical studies featuring anti-VEGF treatment investigate its efficacy in combination with hypofractionated radiotherapy in patients with recurrent GBM (ClinicalTrials.gov identifier NCT01730950). The preliminary results found improved PFS in the group receiving the combination therapy, but the OS was not improved. Furthermore, a variety of resistance mechanisms in tumor vascularization and the tumor microenvironment were observed, thus underlining the urgency of developing new effective targeted therapies [50,141,142]. Apart from the classical VEGFR/VEGF signaling, chemokines are also involved in regulating angiogenesis [37,39,79]. Chemokines and their respective receptors can be divided into proangiogenic and angiostatic subgroups. Among the proangiogenic chemokines are CCL2, CXCL2, CXCL4, CXCL8, CXCL9, CXCL10 and CXCL12 [39,79]. However, CXCL9 and CXCL10 alongside CXCL4 can also hinder angiogenesis [39,79]. CXC chemokines mainly mediate their proangiogenic activity through CXCR2 [62,63] while the angiostatic functions are mediated through CXCR3 [39,79].

CXCR2 and its ligands CXCL2 and IL-8 are overexpressed in GBM and associated with a reduced OS [49,50,67]. Angara et al. found CXCR2^+^ vessel-like structures, which account for a process called vascular mimicry (VM), in GBM patients [50]. VM occurs when tumor cells transdifferentiate and acquire an endothelial-like phenotype to form blood vessels independent of endothelial cells [143,144]. In their study, Angara et al. demonstrated that these VM structures were upregulated after AAT with VEGF pathway inhibitors in an orthotopic mouse model [50]. It has been shown that VM structures are dependent on CXCR2 expression in GBM patients [50,60], therefore, Angara et al. chose to combine AAT with an CXCR2 antagonist to prevent the development of VM [50]. Blockage of CXCR2 signaling with SB225002, a CXCR2 antagonist, significantly reduced tumor growth and CXCR2^+^ VM structures in vivo [50].

**Figure 3:**
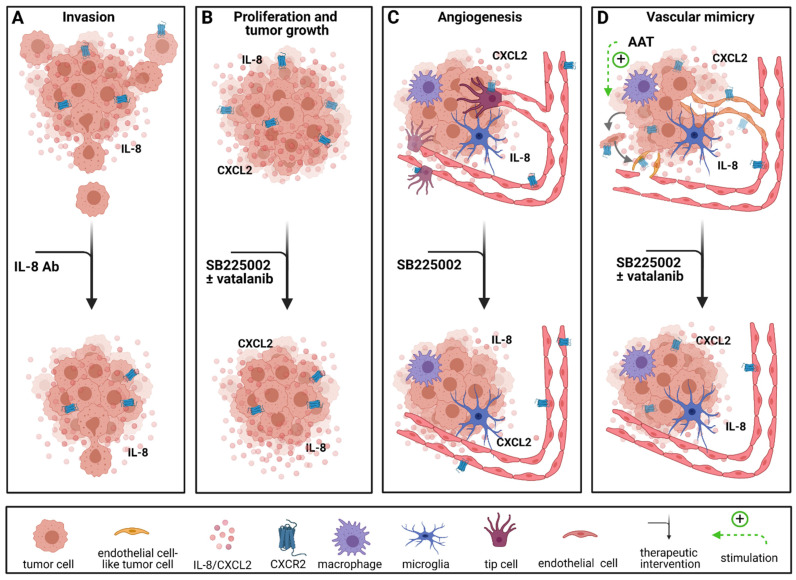
Therapeutic interventions targeting the CXCR2 signaling pathway. (**A**) CXCR2 signaling has been shown to be relevant for glioma invasion which was inhibited by treatment with an IL-8 antibody in vitro. (**B**) Proliferation and tumor growth are reduced by anti-CXCR2 treatment alone and in combination with anti-VEGFR treatment in vivo. (**C**) Anti-CXCR2 treatment also reduces angiogenesis in vitro and in vivo. (**D**) CXCR2 signaling leads to vascular mimicry which is also induced by antiangiogenic treatment and can be inhibited by anti-CXCR2 treatment alone and in combination with anti-VEGFR treatment in vivo. AAT = antiangiogenic treatment with VEGF/VEGFR pathway inhibitors. Created with BioRender.com.

Similar effects were observed in an immunocompetent mouse model where the application of SB225002 led to reduced vessel density, inhibition of tumor growth, reduction in the tumor volume by about 50% and decreased infiltration of TAMs in vivo [68]. Furthermore, SB225002 reduced proliferation of glioma cells and murine endothelial cells in vitro [68] as well as chemokinesis and angiogenesis in a 3D spheroid-based in vitro model with primary human brain endothelial cells [49] (Figure 3).

CXCR4 and CXCR7 alongside other proangiogenic molecules are overexpressed in GBM [52,55,64]. CXCR4-mediated angiogenesis in GBM has been the subject of investigation for almost two decades. CXCL12 and CXCR4 are upregulated by VEGF in vitro [145]. However, CXCL12 and CXCR4 themselves upregulate *VEGF* expression [39,146], which creates an ongoing loop in the VEGF and CXCR4 signaling cascades in GBM. Furthermore, it has been shown that CXCR4 expression is enriched in tip cells [98]. Several studies have also shown that antiangiogenic treatment with bevacizumab and B20, both VEGF inhibitors, as well as with VEGFR inhibitors leads to an elevation of CXCL12 and CXCR4 in vitro and in vivo in a GBM rat model as well as in a PDX mouse model [64,147]. Additionally, CXCR4/CXCL12 are responsible for the homing of endothelial progenitor cells to the tumor bed and thus for initiating vasculogenesis [39].

In an in vitro study with U87 glioblastoma cells, another important crosstalk was evaluated. There, the activation of CXCR4 by CXCL12 led to the protein expression of IL-8 [148]. Nordy, a chiral compound of nordihydroguaiaretic acid and lipoxygenase inhibitor, significantly reduced tumor cell proliferation in vitro and tumor growth in an orthotopic GBM mouse model [148]. Furthermore, Nordy also reduced protein and gene expression of VEGF and IL-8 in vitro, whereas CXCR4 was only decreased on protein level [148].

Apart from its combined effect with the standard-of-care, NOX-A12 targeting CXCL12 exhibited antiangiogenic properties in a rat model [64]. There, combined therapy with a VEGF inhibitor, bevacizumab or B20, led to reduced TAM recruitment and enhanced antitumoral efficacy [64].

As mentioned above, AMD3100, a well-known CXCR4 inhibitor, reduced vessel density and tumor growth in both rat and mouse glioma models [94,149,150]. The effect of AMD3100 in comparison to VEGF pathway inhibitors has been extensively researched in preclinical in vivo models [94,149,150]. These studies have found that AMD3100 exceeded the classical AAT regarding tumor growth rate reduction [94,149,150,151], blood vessel density [149,151] and other vasculogenic parameters [94,150,151]. Kioi et al. found that radiotherapy, one of the pillars of the GBM standard-of-care therapy, triggers the recruitment of BMDCs into the tumors [94]. There, BMDCs restored the radiation-damaged vasculature mediated through the CXCR4/CXCL12 axis by interaction with HIF-1 [94]. Blocking CXCR4 hindered BMDCs from migrating to the tumor after a single dose or fractionated doses of irradiation, thus leading to tumor volume reduction by inhibiting tumor vascularization [94]. In a GBM PDX mouse model, AMD3100 did not only reduce the recruitment of bone marrow-derived endothelial cells, but also of CD68^+^ myeloid cells, compared to vatalinib [152]. It is not surprising that the remarkable success of AMD3100 was accelerated by the combination with anti-VEGF/VEGFR agents [147]. Furthermore, combining anti-VEGF/VEGFR treatment with AMD3100 led to an increased survival of tumor-bearing mice compared to both single strategies and the control group [147]. An additional effect was also observed when pretreating PDX rats with WBIR two weeks prior to tumor cell implantation or pretreatment with AMD3100 followed by anti-VEGFR2 treatment with vatalinib [151]. Vatalinib increased tumor growth and vascular parameters [150,151] which could be significantly reduced by pretreatment with WBIR and AMD3100 [151]. However, the results from a pilot clinical study that mainly enrolled high-grade glioma (HGG) patients who did not receive surgical debulking were not promising [153]. Although the combination of bevacizumab with AMD3100 was well-tolerated, PFS and OS were not significantly improved compared to sole bevacizumab therapy [153,154]. Nevertheless, these results must be interpreted cautiously as the study cohort was very diverse and the information on prior treatments was not provided.

Other CXCR4 antagonists, for instance, PRX177561 and POL5551, have not been extensively studied in combination with AAT. However, their efficacy seems to be comparable to AMD3100. PRX177561 reduced tumor cell proliferation and migration and induced apoptosis in vitro [155]. Furthermore, OS of tumor-bearing mice was prolonged by PRX177561 in an orthotopic mouse model [155]. Gravina et al. then investigated the effect of PRX177561 in combination with sunitinib, a multikinase inhibitor, and bevacizumab. Combining PRX177561 with bevacizumab limited tumor growth and prolonged disease-free survival and OS in subcutaneous and orthotopic xenografts [156]. Moreover, combined therapy with POL5551 and B20-4.1.1, a VEGF antagonist, reduced glioma invasiveness and vascular density compared to sole treatment with B20-4.1.1 in two individual orthotopic glioma mouse models [157].

The combination of VEGF/VEGFR pathway inhibitors with CXCL12/CXCR4 inhibitors showed promising results, making this therapeutic concept an interesting approach that could increase patient survival. However, CXCR4 expression is variable. Therefore, as demonstrated by Dono et al., it is essential to stratify patients according to their CXCR4 expression, as only a subgroup of patients would benefit from anti-CXCR4 therapy [89].

A very different approach was presented by Egorova et al. These authors developed a modular peptide “L1” which was labelled with a CXCR4 ligand to target CXCR4-expressing tumor and endothelial cells [158]. Therefore, L1 did not directly interfere in the CXCR4/CXCL12 axis. However, the authors utilized CXCR4 expression within gliomas to target VEGF mediated angiogenesis. In their study, L1 carried a small interfering RNA (siRNA) silencing *VEGFA* gene expression [158]. With this delivery system, *VEGF* expression in human GBM cells and human endothelial cells significantly decreased in vitro [158]. Furthermore, protein expression of VEGF as well as migration of endothelial cells were significantly decreased in vitro [158].

It is known that CCL2 attracts TAMs [159] which release important proangiogenic factors such as VEGF, CXCL2 and IL-8 [41,42,160]. We recently demonstrated that the infiltration of TAMs was reduced by 30% in an immunocompetent CCR2 knockout mouse model [48]. Additionally, TAMs from tumor-bearing CCR2 knockout mice expressed a reduced amount of *Vegf*, and the tumor vasculature was less leaky and more mature, underlining the importance of CCR2 signaling in GBM angiogenesis [48]. Using the CCL2 inhibitor mNOX-E36 in combination with bevacizumab, Cho et al. confirmed these results by showing a distinct reduction of CD68^+^ monocytes, proliferation and vessel density in a GBM rat model [160]. Although the tumor volume increased in all the groups with time, a significant tumor volume ratio and tumor-doubling time reduction in the group with combined mNOX-E36 and bevacizumab were observed, compared to sole bevacizumab treatment [160]. Furthermore, a survival study in immunocompetent mice showed enhanced long-term survival of mice receiving the combination therapy with mNOX-E36 and bevacizumab compared to bevacizumab alone [160].

### 2.4. Immunotherapeutic Approaches Involving Chemokine Signaling

Inflammatory stimuli are broadly mediated by the chemokine network. Chemokines are involved in autoimmunity, allergy, chronic inflammatory disease and cancer [37]. Apart from their protumoral, proangiogenic and proliferative functions in GBM, they also attract a variety of immune cells including Th1 cells, CD8^+^ T cells, NK cells, granulocytes and TAMs [34,37,39,64]. Among the immunotherapeutic approaches in preclinical and clinical studies in GBM are peptide vaccines, dendritic cell (DC)-based vaccines, adoptive T cell therapy, checkpoint inhibitors and oncolytic virotherapy [22,23,161,162]. As chemokines are involved in the tracking and migration of immune cells [34,37,39,64], they contribute to the efficacy of these immunotherapeutic approaches. Nevertheless, GBM is characterized by its highly immunosuppressive nature which has been described in detail elsewhere [163,164]. In short, multiple factors contribute to the immune-privileged environment of GBM, namely, the blood–brain barrier, the absence of classical lymphatic vessels, immunosuppressive cells within the tumor microenvironment, e.g., regulatory T cells, protumoral TAMs and immunosuppressive factors released by glioma cells [164].

Fighting tumors with genetically engineered immune cells has been a challenging task in cancer research. A promising approach in cell-based tumor therapy is the use of chimeric antigen receptor (CAR) T cells which, upon migration to the tumor, fight it by activating multiple immunological responses [165]. However, major pitfalls of CAR T therapy in GBM are, the immunosuppressive tumor microenvironment and tumor heterogeneity, as well as the lack of migration and persistence of these cells within the tumor [166]. IL-8 exerts its functions through CXCR2, but also through CXCR1 [39]. As high levels of IL-8 are to be expected within the GBM tumor microenvironment, Jin et al. designed CAR T cells with both CXCR1 and CXCR2 receptors [166]. In their U87 glioma mouse model, CAR T cells with both CXCR1 and CXCR2 showed enhanced migration to the tumor and decreased tumor growth compared to the control groups [166]. Moreover, OS of tumor-bearing mice was significantly improved under therapy with CAR T CXCR1 and CXCR2 [166].

CXCR3 is a pivotal receptor in neuroinflammatory and neurodegenerative diseases [37,39,167]. Among its various functions are regulation and activation of infiltrating and resident immune cells in the brain [34,37,167,168]. The expression of CXCR3 ligands CXCL9/CXCL10/CXC11 and CXCL4 is induced by interferons. However, these chemokines then recruit CXCR3-expressing Th1 cells and NK cells which, upon stimulation, produce more interferons, specifically, interferon-γ, thus creating an ongoing loop [39]. Blocking CXCR3 with NBI-74330 in tumor-bearing wildtype and *Cxcr3ko* mice prolonged the median survival of both groups but had no impact on tumor-infiltrating microglia and lymphocytes [169]. Standard anti-inflammatory drugs are mostly cyclooxygenase-2 (COX-2) inhibitors that block the production of prostaglandin E2 (PGE2). As PGE2 induces the expansion of myeloid-derived suppressor cells which promote gliomagenesis, Fujita et al. hypothesized blocking COX-2 would suppress glioma development [170]. COX-2 inhibition resulted in *CCL2* reduction and upregulation of *CXCL10* [170,171]. Furthermore, the tumors were infiltrated by a higher amount of antitumoral CD8^+^ cytotoxic T lymphocytes, while the number of tumor-promoting MDSCs was decreased [170]. Additional blocking of CD40 further prolonged survival of tumor-bearing mice [171]. As described above, GBM is characterized by its immunosuppressive microenvironment [163,164]. Furthermore, Chongsathidkiet et al. discovered a mechanism that hinders T cells from infiltrating GBM tumors [172]. As CTLs exert many antitumoral functions, higher infiltration of these specific T cells is anticipated [163]. In another study, utilizing a GSC-bearing malignant glioma mouse model, Shono et al. demonstrated a dose-dependent antitumoral effect of COX-2 inhibition that, in contrast to the results obtained by Fujita et al. and Kosaka et al., resulted in a reduction of *CCL2, CXCL10* and *CXCR3* [173]. Furthermore, COX-2 inhibition led to NF-κB signaling-dependent downregulation of *CCL2* and *CXCR3*, but not of *CCR2* or *CXCL10* [173]. Moreover, viability of GSC was decreased following silencing of *CCL2* [173]. These findings are somewhat controversial. All three studies analyzed *CXCL10* expression on gene expression levels; however, the used mouse models were different [170,171,173]. While all the studies used C57BL/6 mice, tumor induction or inoculation, respectively, differed. Fujita et al. induced gliomas by DNA transfection of protooncogenes, while Kosaka et al. inoculated GL261 cells and Shono et al. inoculated a GSC line into the mouse brains [170,171,173]. Another important factor is that none of the studies differentiated between CXCR3-A- and CXCR-B-mediated processes, which are known to have opposite functions in GBM [80,81,82,87].

Alternative approaches are cell-based therapies and vaccinations. Over a decade ago, Fujita et al. demonstrated the relevance of CXCL10 for DC-based vaccines in GBM [174]. In their study, intramuscular application of DC vaccines loaded with a glioma-associated antigen (GAA) suppressed intratumoral regulatory T cells and induced antigen-specific antitumoral CTLs [174]. Suppression of intratumoral Tregs is beneficial as Tregs within the GBM microenvironment often exert tumor-supporting functions and thus contribute to GBM progression as described in detail by Wang et al. [163]. The DC vaccine led to prolonged survival of glioma-bearing mice, which was further enhanced by intratumoral injection of the vaccine [174]. However, the vaccine did not exert an antitumoral effect using *CXCL10ko* DCs, thus underlining the importance of CXCL10 in vaccine efficacy [174]. In the same year, Jiang et al. combined glioma lysate-pulsed DCs with CXCL10 in a glioma mouse model [175]. This combination was injected into the tumor, resulting in enhanced antitumoral activity via upregulation of apoptotic tumor cells, decreased proliferation and neovascularization as well as an increase in CTLs [175]. Therefore, it is not surprising that numerous cell-based vaccines were developed in the past years. Comprehensive reviews on dendritic cell vaccines in clinical phase I, II and III studies were recently published by Srivastava et al. [176] and Yan et al. [177]. Another study using a recombinant parvovirus vaccine to deliver CXCL10 and TNF-α, showed a synergic antitumoral effect of both proinflammatory molecules in a GBM mouse model [168].

De Waele et al. proposed a combination of poly(I:C), a synthetic analog of double-stranded RNA and inducer of interferons, with PD-L1 blockade [178]. Anti-PD-L1/PD-1 treatment showed major treatment advances in several human tumors [179]. However, phase III clinical studies of anti-PD-1 monotherapy failed to prolong OS of GBM patients [180] (CheckMate 498; ClinicalTrials.gov identifier NCT02617589). Treatment of glioblastoma cells with poly(I:C) led to enhanced attraction of CTL and, to a lesser extent, of Th cells via CXCR3 and CCR5 signaling by inducing a proinflammatory secretome in vitro [178]. Combined treatment with poly(I:C) and anti-PD-L1 reinforced immune activation in primary GBM cells [178].

It has been demonstrated that NK cells infiltrate gliomas to a lesser extent than other leukocytes under normal circumstances [181]. Müller et al. developed genetically engineered NK cells expressing a chimeric antigen receptor (CAR) targeting EGFRvIII^+^ glioblastoma cells. Infusion of these modified NK cells hindered tumor growth and increased the median survival of subcutaneous tumor xenografts in mice [182]. Due to the promising results, investigators added CXCR4 to the genetically engineered EGFRvIII-specific NKs, thus aiming to target CXCL12-expressing cells. Upon administration of EGFRvIII–CXCR4-specific NKs, tumor reduction and improved OS were achieved, which significantly exceeded the effect of only EGFRvIII-specific NKs [182]. Other immune cells are predominantly found in GBM, e.g., TAMs, which make up about half of the cellular components in GBM [14,127]. Therefore, targeting myeloid cells with anti-CXCR4 treatment is a reasonable concept. The combination of anti-CXCR4 with anti-PD-1, a well-known checkpoint inhibitor, showed promising results [183]. There, animals receiving the combination therapy had a survival benefit compared to the monotherapy arms and, additionally, had a lower number of immunosuppressive infiltrating leukocytes [183]. Furthermore, the combination of anti-CXCR4 and anti-PD-1 led to a significant decrease in the CD4^+^/CD8^+^ ratio and additionally increased levels of circulating inflammatory antitumoral cytokines [183]. Both immunotherapeutic approaches seem to be promising novel therapies that should be investigated in detail.

**Figure 4:**
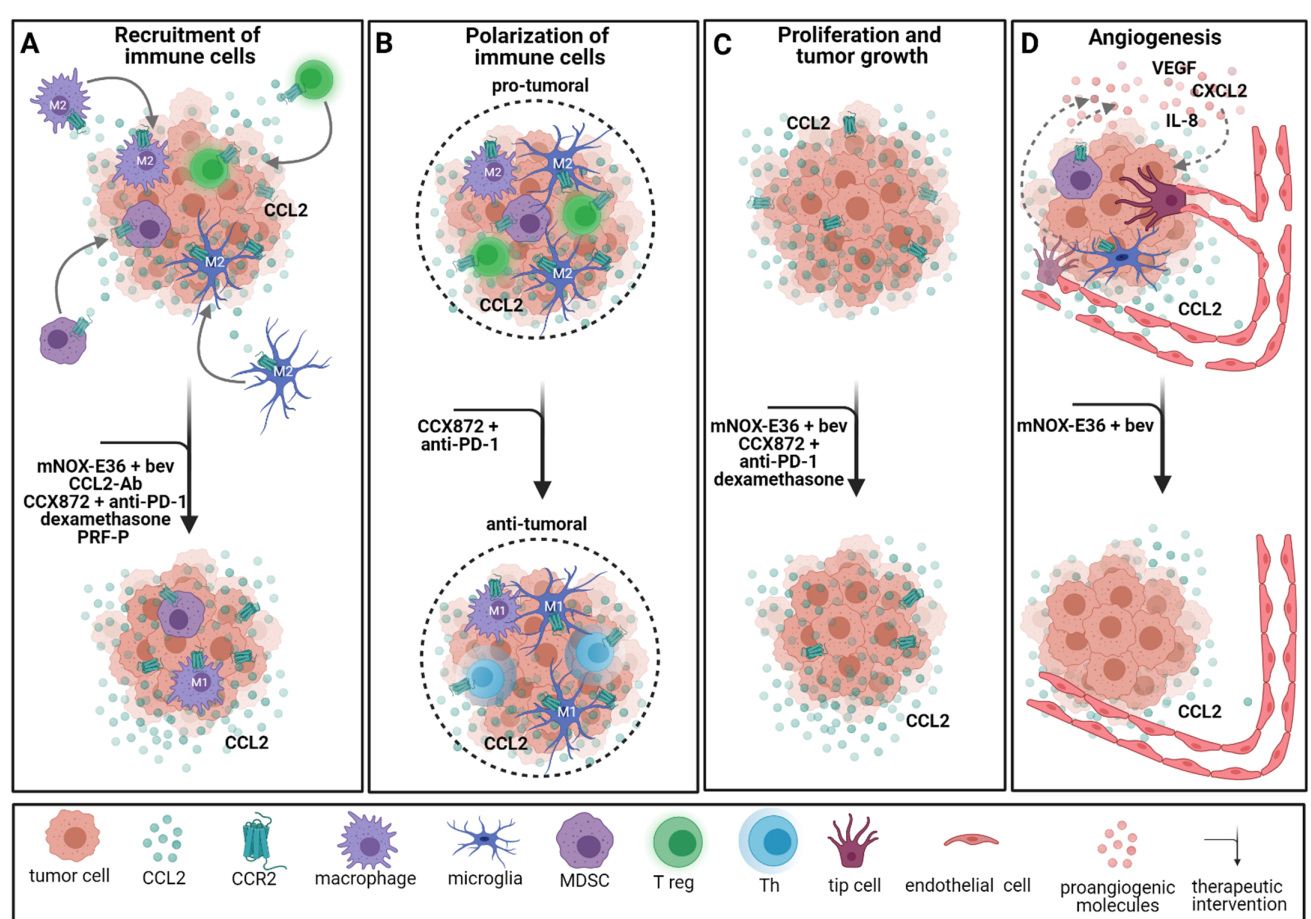
Therapeutic interventions targeting the CCR2 signaling pathway. (**A**) Inhibition of CCR2 signaling alone and in combination with other immunotherapies reduces the recruitment of immune cells to the tumor. (**B**) Polarization of immune cells is dependent on CCR2 signaling. When CCR2 is blocked, TAMs exhibit an antitumoral (M1) phenotype, and tumors are infiltrated by more functional T helper (Th) cells in vivo. (**C**) Proliferation and tumor growth decrease after administration of anti-CCR2 treatment with bevacizumab or other immunotherapies. Dexamethasone also decreases proliferation and tumor growth mediated by CCL2 in vivo. (**D**) Angiogenesis is hindered by combined treatment with a CCR2 inhibitor and bevacizumab in vivo. Additionally, blocking CCR2 leads to reduced TAM infiltration within the tumor. Ab = antibody; bev = bevacizumab; PRF-P = platelet-rich fibrin patch. Created with BioRender.com.

CCR2/CCL2 signaling is known for its proangiogenic and proinflammatory properties. CCL2 recruits regulatory T cells [184,185], NK cells [186] and monocytes, including TAMs [159,187]. However, CCR2 splice variant A can also be found in the cytoplasm of neoplastic cells [188]. Interestingly, Tregs isolated from GBM patients expressed higher levels of CCL2 and its alternative receptor CCR4 than Tregs from healthy controls [184]. CCR2/CCL2 signaling is responsible for the infiltration of both TAMs and Tregs, which often promote immunosuppressive functions facilitating tumor growth [185,189,190]. Therefore, the CCR2/CCL2 axis serves as a viable target for immunotherapies in GBM treatment. Vasco et al. showed that Tregs isolated from GBM patient blood samples migrate towards the GBM cell-conditioned medium in vitro [191]. Compared to the standard medium, Treg migration was significantly increased. Adding a CCL2 antibody to the conditioned medium blocked the migration of Tregs. Later, Panek et al. confirmed the importance of CCR2 signaling in Treg migration [185] and additionally showed that an autologous platelet-rich fibrin patch (PRF-P) suppressed the recruitment of Tregs to tumor-bearing mice [185]. This effect can be explained by increased secretion of the soluble CD40 ligand (sCD40L) by thrombocytes, as blocking sCD40L led to unaltered migration of Tregs [185]. Blocking CCL2 in vitro potentiated the inhibitory effect of PRF-P on Tregs in coculture with GL261 cells [185]. As demonstrated by our group, *Ccr2* knockdown led to a decreased number of TAMs in a GL261 mouse model [48]. Dexamethasone, commonly used to decrease the tumor-surrounding edema, has been shown to alter systemic immunity and especially specific monocyte phenotypes in GBM patients [192]. In a retrospective study of GBM patients accompanied by intravital imaging of tumor-bearing mice, Alieva et al. showed that biopsy-like injuries induce migration and proliferation in GBM. This effect is mediated through CCL2-dependent recruitment of macrophages and can be inhibited by the administration of dexamethasone [193]. Furthermore, dexamethasone treatment prior to biopsy hindered biopsy-induced tumor progression in GBM patients with multifocal tumors [193]. A novel approach is the combination of CCR2 blockade with anti-PD-1 checkpoint inhibitors. As described above, MDSCs are known to manipulate the tumor microenvironment and facilitate tumor growth. CCR2 signaling is believed to play an important role in this process [194]. Combining CCR2 blockade (CCX872) with anti-PD-1 treatment significantly enhanced survival of tumor-bearing mice and reduced MDSCs within the tumor while the fraction of functional T cells was increased [194] (Figure 4).

The less investigated CXCR6/CXCL16 signaling pathway has recently shown to be a feasible target in GBM treatment. While CXCL16 is widely expressed by glioma and glioma stromal cells in vitro and in situ, CXCR6 has been shown to likely be restricted to highly proliferative glioma stem cells [54,195]. Via CXCR6, CXCL16 induced migration and invasion of glial precursor cells in vitro [196]. Lepore et al. confirmed these findings and showed that CXCR6 signaling, mediated by CXCL16, led to tumor cell growth, invasion and migration in vitro and in vivo [66]. In their study, CXCL16, released by glioma cells, drove TAMs towards an anti-inflammatory and thus protumoral phenotype in vitro [66]. However, Hattermann et al. showed that freshly isolated TAMs from GBM tissues express both M1- and M2-specific markers [197]. In their orthotopic GBM mouse model, Lepore et al. compared *Cxcr6* knockout (*Cxcr6ko*) mice to wildtype mice and found that *Cxcr6ko* mice had a distinct prolonged survival [66].

## 3. Conclusions

The chemokine network is a crucial part of the tumor microenvironment in GBM, and chemokine signaling is involved in many tumor-promoting processes. Furthermore, targeting chemokine signaling is a novel therapeutic approach that has shown promising results in preclinical studies in vitro and in vivo. Nevertheless, to date, only agents targeting CXCR4/CXCR7/CXCL12 signaling have been evaluated in clinical studies, showing encouraging results. Thus, based on the promising preclinical studies, new chemokine-targeting therapies should be evaluated in clinical trials, assessing treatment efficacy, PFS and patient OS. As we have learned from previous studies, combining targeted therapies with the standard-of-care, established antiangiogenic treatment or immunotherapeutic approaches should be considered as most effective. These combined therapies could help improve patient OS and overcome therapy resistance in GBM treatment.

## Data Availability

Not applicable.
